# Editorial to Special Issue—Research on Isolation and Intelligent Detection Methods of Foodborne Pathogens

**DOI:** 10.3390/foods11091213

**Published:** 2022-04-22

**Authors:** Daohong Zhang, Jianlong Wang

**Affiliations:** College of Food Science and Engineering, Northwest A&F University, 22Xinong Road, Xianyang 712100, China; zhangdh@nwsuaf.edu.cn

Foodborne illnesses pose a significant threat worldwide to public health. Foodborne pathogens and/or their metabolites are among the prominent important causes of foodborne diseases. In this Special Issue, two articles focused on the detection of foodborne pathogens in foods: one focused on the ultrasensitive quantity detection of *Salmonella Typhimurium* (*S. Typhimurium*) in milk [1], and the other dealing with the viability detection of five common foodborne bacteria in apple juice [2]. Two articles focused on the detection of fungal metabolites: one based on the simultaneous detection of aflatoxin B1 and ochratoxin A [3], and the other on the detection of aflatoxin B1 [4]. Two articles focused on the antibacterial activity of chitosan–nano-ZnO composite films and Thymoquinone against spoilage bacteria and foodborne pathogenic microbes, as well as bacillus cereus and its spores [5,6]. One article investigated the effects of 405 ± 5-nm LED illumination on the environmental stress tolerance of *Salmonella Typhimurium* in sliced beef [7]. One article reviewed rapid detection methods of *Salmonella* in foods [8].

*Salmonella Typhimurium*, which is a widely distributed foodborne pathogen, is tolerant of various environmental conditions [7]. The contamination resulting from the spread of *Salmonella* is a significant public health problem globally; it can cause gastroenteritis, typhoid fever, and even mortality in humans and animals through food chain transmission. To control the contamination and harm of S. Typhimurium in foods, Guo et al. [7] explored the effect of illumination with 405 nm light-emitting diodes (LEDs) on the resistance of *S. Typhimurium* to environmental stress. They first illuminated *S. Typhimurium*-contaminated beef slices under 405 nm LEDs (18.9 ± 1.4 mW/cm^2^) for 8 h at 4 ℃, with another portion of contaminated beef placed in darkness at 4 ℃ as a control. Then, in turn, they exposed the illuminated and non-illuminated beef to thermal stress, oxidative stress, acid stress, and bile salts. Their results showed that, compared with the control group, *S. Typhimurium* in beef slices treated by 405 nm LED irradiation had a reduced survival rate and resistance to these treatments. They explained this phenomenon by demonstrating that the LED illumination could downregulate the transcription of genes related to acid and heat stress resistance. Therefore, according to their experimental results, 405 (±5-nm) LED illumination has application potential in food processing, storage, and transportation in order to control *Salmonella* contamination [7]. Unfortunately, their study did not investigate the inhibition and lowering effects of LED illumination on the survival rate and tolerance to severe environments to other pathogenic bacteria, especially for common foodborne pathogens. This may be a future research direction for their group.

In addition to LED illumination, the use of antimicrobial preservatives is also a common antibacterial strategy. Chitosan is widely used as a natural preservative for fruits and vegetables. To address the issues of the poor mechanical and water resistance properties of common chitosan, Li et al. [5] prepared chitosan–nano-ZnO composite films and assessed the antibacterial activity of these films against selected microorganisms. Using a disc diffusion test, they concluded that the prepared composite film had the strongest antibacterial activity against *A. acidoterrestris*, *S. aureus*, *E. coli*, and *Salmonella*. In a 15-day preservation study, their chitosan composite films exhibited good antibacterial properties by inhibiting the growth of microorganisms on the surface of cherry tomatoes, which is helpful in the prolongation of the shelf life of tomatoes [5]. Additionally, another study also investigated the antibacterial activity of a natural antimicrobial preservative [6]. Bacillus cereus (*B. cereus*) is an important Gram-positive spoilage foodborne pathogenic bacterium in the dairy industry. After contaminating food, *B. cereus* will produce enterotoxins and vomitoxin, subsequently causing vomiting and severe diarrhea in consumers. Wang et al. [6] evaluated the activity of thymoquinone ((TQ) a natural active substance) against *Bacillus cereus* and its inhibitory effect on *B. cereus* spore germination. The bacteriostatic mechanism of TQ is that it can obviously reduce the intracellular ATP concentration of *B. cereus.* Then, the low ATP concentration will cause the depolarization of the cell membrane, increase the intracellular reactive oxygen species level, impair the cell morphology, and destroy proteins or inhibit the protein synthesis of the bacterial cell. In this study, the minimum inhibitory concentrations (MICs) and minimum bactericidal concentrations (MBCs) of TQ against eight *B. cereus* strains were between 4.0 and 8.0 µg/mL [6].

In addition to various antibacterial and sterilization methods, rapid detection techniques are another approach to controlling and preventing the harm caused to consumers by foodborne diseases as a result of foodborne pathogenic bacteria. Gao et al. [1] detected *S. Typhimurium* in milk by using an aptamer–magnetic separation and a multifold Au nanoparticle (AuNP)-based lateral flow assay (LFA). For the detection of *S. Typhimurium*, their method had an excellent linear relationship between 8.6 × 10^2^ and 8.6 × 10^7^ CFU/mL and a supersensitivity with a limit of detection (LOD) of 8.6 CFU/mL in pure culture [1]. In actual samples, the visual LOD of their method was 4.1 × 10^2^ CFU/mL, without nucleic acid amplification and pre-enrichment [1]. It is possible that their LFA method could also validate the bacteriostasis effectiveness of S. Typhimurium in some studies, such as the validity of the above research conducted by Guo et al. [7], Li et al. [5], and Wang et al. [6].

In addition to the above-mentioned rapid detection of foodborne pathogenic bacteria using classical lateral flow immunoassay, the following study proposes another detection concept via the rapid colorimetric testing of five common foodborne bacteria in apple juice. Apple juice may be contaminated by foodborne microorganisms due to its high content of glucose. Zhang et al. [2] prepared a cascade composite nanozyme, GOx@GA-Fe (ii), by combining glucose oxidase and peroxidase activities, and then developed a colorimetric method to detect the viability of five common foodborne bacteria (*Escherichia coli*, *Staphylococcus aureus*, *Salmonella typhimurium*, *Listeria monocytogenes*, and *Enterobacter sakazalii*) in apple juice samples. As the prepared nanozyme could catalyze glucose by TMB colorimetry, it could detect bacteria in actual samples through glucose consumption. If there were living bacteria in the apple juice, they would consume the glucose contained in the juice; then, after the addition of TMB, no glucose or just a low content glucose would be catalyzed, and the sample solution would not change color. On the contrary, if there were dead bacteria in the apple juice, the color of the sample solution would become blue. Therefore, in this work, the presence of living bacteria decreased the absorbance of the sample solution, whereas dead bacteria had no obvious effect on absorbance [2]. The authors declared that, compared with most detection methods, their method did not require precision instruments, and had a high detection accuracy over wide temperature and pH ranges. However, unfortunately, the analytical principle of this study found that their strategy could only be used to measure bacterial viabilities in samples with a high glucose content, and will have no monitoring efficiency in the bacterial contamination of samples with no or a very low glucose content. In contrast, the application scope of the method developed by Zhang et al. [2] is much narrower than that of the LFA established by Gao et al. [1], but the previous method can simultaneously monitor several different bacteria, while the latter method can only detect *S. Typhimurium*, unless other test lines are coated at the same time, aiming at more target pathogenic bacteria.

Fungi, another kind of foodborne microorganism, can also contaminate food and cause foodborne illnesses by generating toxic secondary metabolites. Mycotoxins are highly stable and heat-resistant metabolites produced by certain fungi under suitable conditions. Zhao et al. [3] and Tang et al. [4] separately detected mycotoxins. Both aflatoxin B1 (AFB 1) and ochratoxin A (OTA) are highly toxic and carcinogenic mycotoxins. Zhao et al. developed an immuno-chromatographic strip test for the simultaneous quantitative detection of AFB 1 and OTA in spices [3]. The detection limits of their test strips for AFB 1 and OTA in Chinese prickly ash, pepper, chili, cinnamon, and aniseed were 3 and 5 µg/kg, respectively. The detection results of their strip tests in actual samples were consistent with those of high-performance liquid chromatography (HPLC) analysis, with a false-positive rate and false-negative rate of 2% and 0%, respectively. After testing using strip tests, 17 spice samples (over 10% of the total samples) were found to be contaminated by mycotoxins, and among them, AFB 1 in one pepper sample and OTA in two chili samples exceeded the maximum permissible limit set by the European Union [3]. In contrast to the work of Zhao et al., Tang et al. prepared a portable, low-cost, and user-friendly colorimetric instrument for the colorimetric detection of aflatoxin B1 using ELISA [4]. Their measurement results of AFB 1 in maize and peanut samples showed that the instrument provided equally accurate results when compared to the professional equipment. It is worth mentioning that their instrument is user-friendly, with the ability to calculate and report the final amount of AFB 1 to the operator; inexpensive, with a capitalized cost of approximately RMB 129 or USD 20; and portable, with an easily handheld size for users. The limit of detection (LOD) for AFB 1 standard was determined to be 0.06 µg/L. In the practical application, the instrument could be easily calibrated by using a blank solution and a negative sample free of AFB 1. Although the instrument has considerable promise for the quantitative and cost-effective detection of contaminants in foods, it was only able to measure the samples one by one. In most cases, ELISA tests are performed in a high-throughput manner; then, accordingly, rapid and multi-channel measurements are required for the readout of results. Therefore, in the following study, the authors should strive to improve the multi-channel analysis capability of their instrument [4].

Wang et al. [8] reviewed rapid detection methods of *Salmonella* in foods. In the review, they summarized and described the bioreceptors for *Salmonella*, including the antibody, aptamer, nucleic acid probe, bacteriophage, and lectin. In these bioreceptors, each has its own advantages and limitations, and the antibody has been well recognized as the standard bioreceptor of commercial immunochromatographic lateral flow strips and the enzyme-linked immunosorbent assay (ELISA) kits used in food safety detection [8]. Then, they objectively classified and summarized the current promising rapid detection methods for *Salmonella* in foodstuffs with different signal transductions. Optical sensors (including colorimetric, fluorescence, surface-enhanced Raman spectral (SERS), surface plasmon resonance (SPR), and photothermal detection methods), electrochemical sensors (including voltametric and impedance sensors), and other signal transduction methods (including piezoelectric and magnetic relaxation biosensors) were introduced and summarized. Among them, both the optical and electrochemical biosensors are classical methods for *Salmonella* detection. The optical sensors include a wide variety of types; those that can convert biological and chemical reactions into optical signals through transducers or detection instruments can be categorized as optical sensors. However, electrochemical sensing usually has extremely low limits of detection (LOD) for bacteria. Finally, they underlined the challenges faced in these methods in practical applications and proposed a new perspective for the development of *Salmonella* rapid detection methods. In their opinion, the application obstacles of various rapid detection methods mainly come from three aspects, namely, complex sample pretreatment, low stability, and difficulties of nondestructive testing and in-field application. They present prospects for the future development of rapid detection technologies from the perspectives of miniaturization and automation equipment, the combination of multiple sensing methods, microfluidic technologies, and the combination of information technology and big data analysis. Like these authors, we also believe that more automated, sensitive, portable, and low-cost technologies will be developed in the future by assiduous researchers for the rapid detection of foodborne pathogenic bacteria to provide effective early warnings for the safe consumption of foods.

These articles extended the knowledge on the control and detection of foodborne microbes or their metabolites by:

- Exploring the effects of 405 (±5) nm LED illumination on the environmental stress tolerance of *Salmonella Typhimurium* in sliced beef;
- Assessing the antibacterial activity of chitosan–nano-ZnO composite films against microorganisms;
- Evaluating the antimicrobial activity of thymoquinone against Bacillus cereus and its spores;
- The ultrasensitive detection of *Salmonella Typhimurium* in milk using an enhanced lateral flow immunoassay;
- The cascade detection of bacterial viability in apple juice using a self-assembled nanozyme;
- The simultaneous detection of aflatoxin B1 and ochratoxin A in spices using lateral flow immunoassays;
- Preparing a portable, cost-effective, and user-friendly instrument for the colorimetric detection of aflatoxin B1;
- Providing an overall review of the rapid detection methods for *Salmonella*.
